# Anemia in relation to body mass index and waist circumference among chinese women

**DOI:** 10.1186/1475-2891-12-10

**Published:** 2013-01-11

**Authors:** Yu Qin, Alida Melse-Boonstra, Xiaoqun Pan, Baojun Yuan, Yue Dai, Jinkou Zhao, Michael B Zimmermann, Frans J Kok, Minghao Zhou, Zumin Shi

**Affiliations:** 1 Department of Chronic Disease Control, Jiangsu Province Centre for Disease Control and Prevention, Nanjing, China; 2 Division of Human Nutrition, Wageningen University, Wageningen, The Netherlands; 3 Laboratory for Human Nutrition, Swiss Federal Institute of Technology (ETH) Zürich, Zürich, Switzerland; 4 Discipline of Medicine, University of Adelaide, Adelaide, Australia

**Keywords:** Anemia, Body mass index, Waist circumference, China, Women

## Abstract

**Background:**

This study aimed to investigate the relationship of anemia and body mass index among adult women in Jiangsu Province, China. Data were collected in a sub-national cross-sectional survey, and 1,537 women aged 20 years and above were included in the analyses. Subjects were classified by body mass index (BMI) categories as underweight, normal weight, overweight and obese according to the Chinese standard. Central obesity was defined as a waist circumference ≥ 80 cm. Anemia was defined as hemoglobin concentration < 12 g/dl. Prevalence ratios (PRs) of the relationship between anemia and BMI or waist circumference were calculated using Poisson regression.

**Findings:**

Overall, 31.1% of the Chinese women were anemic. The prevalence of overweight, obesity and central obesity was 34.2%, 5.8% and 36.2%, respectively. The obese group had the highest concentrations of hemoglobin compared with other BMI groups. After adjustment for confounders, overweight and obese women had a lower PR for anemia (PR: 0.72, 95% CI: 0.62-0.89; PR: 0.59, 95% CI: 0.43-0.79). Central obesity was inversely associated with anemia.

**Conclusion:**

In this Chinese population, women with overweight/obesity or central obesity were less likely to be anemic as compared to normal weight women. No measures are required currently to target anemia specifically for overweight and obese people in China.

## Findings

Anemia can increase risk of maternal and child mortality, impair cognitive and physical development in children, and endanger physical performance in adults [1,2]. In China, 20% of preschool children and of non-pregnant women at reproductive age are affected by anemia [3], which can be classified as a moderate public health problem according to world health organization criteria (WHO) [2].

Obesity has been reported to be associated with anemia in adults in some countries [4-10], which may be due to up-regulated hepcidin expression thereby hampering iron absorption [11]. Therefore, obesity could potentially add to the burden of anemia in China, since the country has experienced an alarming increase in obesity-related chronic diseases over the past decade [12]. Therefore, we investigated the relationship between overall and central obesity with anemia among a female Chinese population.

## Method and materials

The study was conducted in Jiangsu Province using a multistage cluster sampling method as described before [13], which was part of a national representative cross-sectional study on Nutrition and Health conducted in 2002. The study was approved by the Human Investigation Review Committee at the National Institute for Nutrition and Food Safety, Chinese Center for Disease Control and Prevention. Written informed consent was obtained from the subject for publication of the data collected. Three towns and six counties were selected based on geographic directions and economic development. Three street/towns were randomly selected from each of the cities and counties. In each town/street, two villages/neighborhoods were further randomly selected. In each village/neighborhood, 30 households were randomly selected. All members in the households were invited to take part in the study. Altogether, 1,537 out of 1,652 women with complete data were included in the survey. Energy and nutrient intake was calculated using three consecutive days of 24-h dietary recall in conjunction with the China Food Composition Table published in 2002 [14]. Anemia was defined as a hemoglobin concentration below 12 g/dl [1]. Women were classified by BMI categories as underweight (BMI < 18.5), normal weight (BMI > 18.5 < 24), overweight (BMI ≥ 24 < 28) and obesity (BMI ≥ 28) according to the Chinese standard. Central obesity was defined as a waist circumference ≥ 80 cm [15]. Low socio-economic status (SES) was defined as an annual income of less than 1,999 Yuan, ‘medium’ as 2,000–4,999 Yuan and ‘high’ as more than 5,000 Yuan.

Variables were presented as percentage or means ± standard deviations (SD) by BMI categories, with chi-square test for categorical variables and ANOVA for continuous variables. Poisson regression was used to estimate the association as prevalence ratios (PRs) between BMI categories, central obesity and anemia controlling for confounders, including age, residence, SES, educational level, and daily energy and iron intake.

## Results

The mean age of the subjects was 46.4 ± 14.7 yrs. The average BMI was 23.6 ± 3.7. Table 1 presents general characteristics of the study population by BMI categories. Compared with underweight subjects, obese/overweight women had higher iron intake. Energy intake and hemoglobin concentration increased over BMI categories. The overall prevalence of anemia was 31.1%. Women with high and medium SES had higher prevalence of anemia than women with low SES (35.4% and 35.9% vs 21.5%, P < 0.001). No difference in prevalence of anemia was found for age, urban/rural and education level groups. Anemia showed a significant decreasing trend with increasing BMI. Compared to normal weight women, overweight and obese women had lower PRs for anemia (PR: 0.72, 95% CI: 0.62-0.89; PR: 0.59, 95%CI: 0.43-0.79). Central obesity was also inversely associated with anemia (PR: 0.75, 95% CI: 0.63-0.89).

**Table 1:** General characteristics of the study population (n = 1,537) by BMI categories

	**Underweight**	**Normal weight**	**Overweight**	**Obese**	**P value**
**n (%)**	**89 (5.8)**	**834 (54.3)**	**424 (27.6)**	**190 (12.4)**	
Age (years) (%)					
< 35	38.2	29.5	16.5	9.5	< 0.001
35-44	13.5	25.4	21.9	21.6	
45-54	13.5	19.9	33.7	26.3	
≥ 55	34.8	25.2	27.8	42.6	
Residence					
Urban city	23.6	22.1	27.4	31.1	0.03
Rural city	76.4	77.9	72.6	68.9	
Socio-economic status					
Low	26.1	32.4	33.9	34.0	0.39
Medium	38.6	31.0	34.8	33.0	
High	35.2	36.6	31.3	33.0	
Education					
Low	50.6	54.9	59.4	65.3	0.002
Medium	27.0	32.3	30.9	21.1	
High	22.5	12.8	9.7	13.7	
Hemoglobin (g/L)^1^	125.0 (14.6)	124.9 (15.4)	128.4 (15.0)	131.9 (15.1)	< 0.001
Energy (kcal/d)	1916 (563)	2124 (582)	2179 (639)	2198 (637)	0.001
Iron (mg/d)	20.0 (7.4)	23.4 (9.6)	24.2 (9.8)	23.8 (9.5)	0.003
Vitamin C (mg/d)	61 (33)	61 (38)	59 (39)	60 (36)	0.886

^1^Mean (SD), all such values; BMI, body mass index. Chi-square test for categorical variables and ANOVA for continuous variables.

## Discussion

In this representative cross-sectional study, we found that both overweight/obesity and central obesity were inversely associated with anemia. The result was consistent with studies from Peru, Egypt, and the US, but not with a Mexican study [4,5]. The level of iron and vitamin C intake may partly explain the discrepancy. In our study, average iron intake ranged from 20 to 24 mg/d, which is in line with the Adequate Intake of 20 mg/d for Chinese women [16]. Although obese/overweight women had a higher intake of iron than underweight women, the average intake of underweight women was still adequate. In contrast, in the Mexican population iron intake was reported to be in the range of 8–9 mg/d [5]. Intake of vitamin C, the most potent enhancer of non-heme iron absorption, was present in sufficient amounts in the diets of Chinese women (60 mg/d), whereas vitamin C intake in Mexican women was low (30 mg/d). It may be that the Chinese diet conveys enough absorbable iron to lower the risk of anemia in contrast to the Mexican diet [6]. Although overweight or obesity in the population may not decrease red-cell survival or impair erythropoiesis, obesity might still result in hypoferremia through hepcidin or other mediators [4].

Waist circumference reflects intra-abdominal fat mass, and is related to cardiovascular diseases in adults [17]. Limited studies have assessed the relationship between central obesity and anemia. Gillum et al. [8] reported a positive association between waist-hip-ratio and serum ferritin. In our study, women with central obesity were less likely to have anemia, consistent with the results with overall obesity.

The main limitation of our study is that we used anemia as an indicator which only represents a part of the complex assessment of iron status. Therefore, we cannot truly distinguish anemia of chronic disease and anemia caused by iron deficiency.

In conclusion, in this study we found an inverse association between overweight/obesity, central obesity and anemia in Chinese women from Jiangsu Province. Our study contributes to the existing knowledge base on the complex association between adiposity and anemia. Inclusion of multiple iron and inflammation markers in future studies could possibly unravel the true meaning of our findings.

## Abbreviations

BMI: Body mass index; PRs: Prevalence ratios; WHO: World health organization; SES: Low socio-economic status; SD: Standard deviations.

## Competing interests

The authors declare that they have no competing interests. The Project was supported by Jiangsu Provincial Health Bureau. Yu Qin is supported by an INREF fellowship from Wageningen University, the Netherlands. There are no financial competing interests.

## Authors’ contribution

YQ, contributed to the design, field work, data collection, analysis and manuscript writing. AMB, corresponding author, contributed to data interpretation and manuscript writing. XP, BY, YD, JZ, and MZ, contributed to the implementation in the field and gave advice on the manuscript writing. MBZ, FJK, and ZS, contributed to the overall scientific overview throughout the study and to manuscript writing. All authors read and approved the final manuscript.
